# Path-based extensions of local link prediction methods for complex networks

**DOI:** 10.1038/s41598-020-76860-2

**Published:** 2020-11-16

**Authors:** Furqan Aziz, Haji Gul, Irfan Uddin, Georgios V. Gkoutos

**Affiliations:** 1 Centre for Computational Biology, University of Birmingham, Birmingham, B15 2TT UK; 2 College of Medical and Dental Sciences, Institute of Cancer and Genomic Sciences, University of Birmingham, Birmingham, B15 2TT UK; 3 Institute of Translational Medicine, University Hospitals Birmingham NHS Foundation Trust, Birmingham, B15 2TT UK; 4 MRC Health Data Research UK (HDR), Midlands, UK; 5 City University of Science and Technology, Peshawar, Pakistan; 6 Kohat University of Science and Technology, Kohat, Pakistan; 7 NIHR Experimental Cancer Medicine Centre, Birmingham, B15 2TT UK; 8 NIHR Surgical Reconstruction and Microbiology Research Centre, Birmingham, B15 2TT UK; 9 NIHR Biomedical Research Centre, Birmingham, B15 2TT UK

**Keywords:** Applied mathematics, Computer science, Information technology

## Abstract

Link prediction in a complex network is a problem of fundamental interest in network science and has attracted increasing attention in recent years. It aims to predict missing (or future) links between two entities in a complex system that are not already connected. Among existing methods, local similarity indices are most popular that take into account the information of common neighbours to estimate the likelihood of existence of a connection between two nodes. In this paper, we propose global and quasi-local extensions of some commonly used local similarity indices. We have performed extensive numerical simulations on publicly available datasets from diverse domains demonstrating that the proposed extensions not only give superior performance, when compared to their respective local indices, but also outperform some of the current, state-of-the-art, local and global link-prediction methods.

## Introduction

The study of complex networks is a relatively new, but rapidly growing field of interdisciplinary scientific research that aims at modelling and analysing real-world complex systems^1,2^. The interest in network science has emerged from the empirical study of networks that are obtained as a result of modelling real-world complex systems^3^. Example of such networks include ecological networks^4^, social networks^5^, transportation networks^6^, and biological networks^7^. A complex network provides a convenient way of representing a complex system where nodes of the network represent entities of the complex system and links represent interactions between entities. However, the process of acquiring networks from complex systems may introduce noise which can result in missing links in a network^8^. To tackle this issue, link prediction has attracted the attention of researchers from a diverse scientific disciplines. It aims to estimate the likelihood of the existence of a link between disconnected nodes based on node attributes, neighbour information, and network structures. The problem is of both theoretical interest and has broad applications. Some of its applications include friend recommendation in social networks such as Facebook^9^, predicting interactions between proteins^10^, product recommendation to users^11^, and drug target interaction prediction^12^.

Motivated by the practical significance of link prediction, numerous link prediction algorithms have been proposed for unweighted networks. Among the various categories of link prediction algorithms, the most popular ones are the structural based similarity indices that are based solely on the structural properties of the underlying complex network. One of the most commonly used structural based similarity indices is the common neighbour^13^, which measures the similarity between two nodes by counting the number of common neighbouring nodes. This method, however, does not take into account the degree information of the two nodes or their common neighbours. To overcome this problem, many variations of common neighbours have been proposed. For example, Adamic-Adar^14^ and resource allocation^15^, that penalise the high-degree common neighbour and perform better than common neighbour in most practical situations. Other local indices include preferential attachment^16^ Jaccard coefficient^17^, Sørensen index^18^, and Salton index^19^. Cannistraci et al.^20^ have combined a local link prediction algorithm with a local community structure to define a new set of link prediction indices, namely the CAR-based indices, demonstrating the application of CAR-based indices in predicting links in brain connectomes. However, these similarity indices are defined for an unweighted and undirected network. In order to predict links in weighted networks, Zhao et. al.^21^ have extended the unweighted similarity indices to weighted ones that can, not only, predict the missing link in a network but also estimate the weight of the missing link. Furthermore, Ghorbanzadeh et al.^22^ have defined a measure, based on common neighbour, that can be applied to directed networks.

The structure-based similarity indices discussed so far are also called local similarity indices (or node dependent similarity indices) as they are based on the information of the immediate neighbours of the two query nodes. An alternative approach to link prediction is to consider the overall structure of the network. Such type of similarity indices are called global similarity indices. Global methods are also sometimes called path-dependent similarity indices since they are generally based on path information between nodes. One example is the Katz index^23^ which considers the set of all paths between the two query nodes. Recently, Yant et al.^24^ and Ahmad et al.^25^ have proposed methods that take advantages of both the local and the global properties of a network by combining common neighbour and distance information to estimate the likelihood of the formation of link between two nodes. Other global similarity indices include hitting (or commute) time^26^, Matrix-Forest Index^27^, Linear Optimization^28^, SimRank^29^, and similarity-popularity based methods^30^.

To provide a tradeoff between accuracy and computational time, quasi-local indices^31,32^ are introduced that consider paths with wider horizon. To that end, Lü et al^33^ have defined local path (LP) index that considers local paths of shorter lengths between the two query nodes. They have empirically shown that the LP index performs better when compared to common neighbour and gives comparable performance to Katz index^23^. They have also demonstrated that LP has low computational time as compared to Katz index. Just like common neighbour, LP index suffers from the problem that it does not take into account the degree information of the two nodes and the nodes on the local paths. In this paper we propose novel global and quasi-local measures that extend the existing local measures and can be used to predict missing link with higher accuracy. The idea is to use the node information on local paths. We commence by providing vectorised implementation of several local structural based similarity indices. For each of those similarity indices, we propose their global and quasi-local extensions. In the experimental evaluation section, we compare the local indices to their global and quasi-local extensions and empirically demonstrate that the global (and quasi-local) indices usually give better performance (but have higher computational cost) as compared to their corresponding local indices. In particular, we show that the difference in performance is significant when the noise in the data is high. We also demonstrate that some of the global indices introduce in this paper outperform the Katz index.

## Overview of link prediction

In this section, we introduce some of the state-of-the-art local link prediction indices. We also present the local path index^33^ and the Katz index^23^, considered as the corresponding quasi-local and global extensions of the common neighbour index^13^. A *network* $G = (V, E)$ is defined as a set *V* of *nodes* and a set *E* of *links*, where $E \subseteq V \times V$. A network is *directed*, if the links it contains are directed, and *undirected* if the links it contains have no direction. A network is termed *weighted*, if the links it contained are assigned different weights. Otherwise, it is termed *unweighted*. A *simple* network is a network where multiple links between nodes as well as links between same nodes (self-loops) are not allowed. In this work, we considered simple, undirected and unweighted networks. The *degree* of a node $v\in V$, represents the number of connections that a node has with other network nodes. We denote the degree of the node *v* by $|\Gamma (v)|$, where $\Gamma (v)$ represents the set of all neighbours of *v*. A *walk* *w* in a network $G = (V,E)$ is defined as a sequence of alternating nodes and edges $v_0,e_1,v_1, e_2, v_2, ..., e_k, v_{k}$ where $v_i\in V$ and $e_i =(v_{i-1},v_i)$. This walk has *length* *k*, where *k* is the number of links in the walk. An *adjacency matrix *provides a compact way of representing a network $G = (V,E)$. It is a square matrix of size $\left| V\right| \times \left| V\right|$, whose (*u*, *v*)th entry is 1 if *u* and *v* are linked and 0 otherwise. The (*u*, *v*)th entry of the $k^{th}$ power of the adjacency matrix , $(A^k)_{uv}$, represents the number of walks of length *k* from *u* to *v*.

We now present some of the commonly used local similarity indices. In the next section we present their global and quasi-local extensions.

*Common Neighbour (CN)*^13^: A common neighbour is a simple but effective measure based on the number of shared neighbours between two nodes. In other words, two nodes are more likely to have a link if they share many common neighbours.
*Adamic Adar (AA)*^14^: AA is a variant of the CN that assigns more weight to neighbours with lower degrees. It captures the notion that neighbours with fewer links are more influential in facilitating the formation of future interactions.
*Resource Allocation (RA)*^15^: RA is defined in a similar way to AA. However, compared to AA, it assigns a lower score to the node pairs whose common neighbours have a high node degree. The only difference in the mathematical representation of RA and AA indices is that the later takes the logarithm of the denominator.
*Sørensen (SO)*^18^: Sørensen Index was proposed to establish equal amplitude groups in plant sociology based on the similarity of species. It is also used to calculate similarities of nodes in complex networks. It is determined by common neighbours of node pairs relative to their sum of individual degrees.
*Salton (SA)*^19^: This measure, proposed by Salton and McGill, is based on cosine angle between rows of adjacency matrix having query nodes *u* and *v*. This index is also called Salton Cosine Index.
*Leicht Holme Newman (LHN)*^34^: This measure gives higher score for node pairs having more common neighbours in proportion to their expected number of neighbours.
*Hub Promoted(HP)*^35^: This index is proposed for quantifying the topological overlap of pairs of substrates in metabolic networks. Here, node pairs adjacent to hub nodes are assigned higher scores.
*Hub Depressed(HD)*^15^: This measure is similar to HP measure but it is affected by higher degree nodes. Any node which has high degree is penalised by this measure.

Table 1 (first column) gives mathematical formulation for each of these local similarity indices. Although local similarity measures can be computed efficiently and perform relatively well, their accuracy cannot generally reach to methods which are based on global information. One typical example of global similarity index is the Katz index which is defined as follows:

*Katz Index* ($Katz_{\beta })$^23^ This index computes the similarity scores, based on the set of paths of different lengths, between two query nodes. The paths are exponentially damped by the length of the path so to assign more weight to shorter paths. Mathematically, this index is defined as follows:

1 $$\begin{aligned} Katz_{uv} = \sum _{l=1}^{\infty }\beta ^l \cdot \left[ \text {path}_{uv}^{\langle l \rangle }\right] , \end{aligned}$$

where $0<\beta <1$ is the parameter that controls the weight of the paths of different lengths. The similarity matrix *S*, whose (*u*, *v*)th entry equals $Katz_{uv}$, can also be computed as $\left( I-\beta A\right) ^{-1}-I$, where *I* represents the identity matrix of size |*V*|.

Since this method is based on the topology of the whole network, therefore it generally outperforms local similarity indices such as CN. The difference is significant when the network is sparse, or when the network has many missing links. However, global indices generally have higher computational time when compared to local indices. In order to provide a good trade-off between accuracy and Complexity, Lü et al.^33^ have introduced path index, which is defined as follows:

*Local Paths (LP)*^33^ This index considers locals paths of shorter length and is generally computed as $A^2+\beta A^3$, where *A* is the adjacency matrix of the network. As with Katz index, $\beta <1$ is set to a small value so that shorter paths get more weights.

Lü et al.^33^ have empirically demonstrated that LP index performs remarkably better than the simple CN index. They have also demonstrated that both Katz and LP indices generally give comparable performances, while the computation time of LP is considerably low than Katz index. Note that CN index, LP index, and Katz index have unified form as all the three indices can be expressed using Eq. (1), where for CN $l=2$, for LP $l=2,3$, and for Katz $l=1,2,...,\infty$. Therefore, both LP and Katz indices can be considered as extensions of Common neighbours to local paths.

## Methods

In this section, we define the global and the quasi-local extensions of some of the most widely used local similarity indices. For each local similarity index, we first give its vectorised implementation. Our goal is to define global indices similar to Katz index that reduce to local indices for smaller values. Additionally, we also propose quasi-local measures of these indices. In the experimental evaluation section of this paper, we demonstrate that the global and quasi-local indices of RA and AA indices generally outperform all the other indices on most of the datasets. However, for the sake of completeness and experiments, we also define the global and the quasi-local extensions for the remaining local similarity indices. These similarity indices are summarised in Table 1, where the first column gives the mathematical definition of the local similarity index and the second column provides a matrix representation of the local index. The global and the quasi-local extension of the respective local similarity index are also given in the third and fourth column of the table respectively. In the remaining of this section, we briefly discuss how the global and quasi-local extensions are obtained.

**Table 1:** Local, Quasi-local, and Global Similarity indices.

Local index	Matrix form	Global extension	Quasi-local extension
$CN(u,v) = \left| \Gamma (u)\cap \Gamma (v)\right|$	$A^2$	$\left( I-\beta A\right) ^{-1}-I$	$A^2 + \beta A^3$
$RA(u,v) = \sum _{w \in \{ \Gamma (u)\cap \Gamma (v) \}}\frac{1}{ \left| \Gamma (w)\right| }$	$AD^{-1}A$	$A\left( I-\beta D^{-1}A\right) ^{-1}-A$	$AD^{-1}A+\beta AD^{-1}AD^{-1}A$
$AA(u,v) = \sum _{w \in \{ \Gamma (u)\cap \Gamma (v) \}}\frac{1}{\log \left| \Gamma (w)\right| }$	$A\left( \log D\right) ^{-1}A$	$A\left( I-\beta (\log D)^{-1}A\right) ^{-1}-A$	$A\left( \log D\right) ^{-1}A+\beta A\left( \log D\right) ^{-1}A\left( \log D\right) ^{-1}A$
$SO(u,v) = \frac{2\left| \Gamma (u)\cap \Gamma (v)\right| }{|\Gamma (u)|+|\Gamma (v)|}$	$2\left( D\left( A^2\right) _{ij}^{-1}+\left( A^2\right) _{ij}^{-1}D\right) _{ij}^{-1}$	$2\left( D\left( \left( I-\beta A\right) ^{-1}-I\right) _{ij}^{-1}+\left( \left( I-\beta A\right) ^{-1}-I\right) _{ij}^{-1}D\right) _{ij}^{-1}$	$2\left( D\left( A^2+\beta A^3\right) _{ij}^{-1}+\left( A^2+\beta A^3\right) _{ij}^{-1}D\right) _{ij}^{-1}$
$SA(u,v) = \frac{\left| \Gamma (u)\cap \Gamma (v)\right| }{\sqrt{|\Gamma (u)|\times |\Gamma (v)|}}$	$D^{-\frac{1}{2}}A^2D^{-\frac{1}{2}}$	$D^{-\frac{1}{2}}\left( \left( I-\beta A\right) ^{-1}-I\right) D^{-\frac{1}{2}}$	$D^{-\frac{1}{2}}A^2D^{-\frac{1}{2}}+\beta D^{-\frac{1}{2}}A^3D^{-\frac{1}{2}}$
$LHN(u,v) = \frac{\left| \Gamma (u)\cap \Gamma (v)\right| }{|\Gamma (u)|\times |\Gamma (v)|}$	$D^{-1}A^2D^{-1}$	$D^{-1}\left( \left( I-\beta A\right) ^{-1}-I\right) D^{-1}$	$D^{-1}A^2D^{-1}+\beta D^{-1}A^3D^{-1}$
$HP(u,v) = \frac{\left| \Gamma (u)\cap \Gamma (v)\right| }{\min (|\Gamma (u)|, |\Gamma (v)|)}$	$\left( \min \left( D\left( A^2\right) _{ij}^{-1},\left( A^2\right) _{ij}^{-1}D\right) \right) _{ij}^{-1}$	$\left( \min \left( D\left( \left( I-\beta A\right) ^{-1}-I\right) _{ij}^{-1},\left( \left( I-\beta A\right) ^{-1}-I\right) _{ij}^{-1}D\right) \right) _{ij}^{-1}$	$\left( \min \left( D\left( A^2+\beta A^3\right) _{ij}^{-1},\left( A^2+\beta A^3\right) _{ij}^{-1}D\right) \right) _{ij}^{-1}$
$HD(u,v) = \frac{\left| \Gamma (u)\cap \Gamma (v)\right| }{\max (|\Gamma (u)|, |\Gamma (v)|)}$	$\left( \max \left( D\left( A^2\right) _{ij}^{-1},\left( A^2\right) _{ij}^{-1}D\right) \right) _{ij}^{-1}$	$\left( \max \left( D\left( \left( I-\beta A\right) ^{-1}-I\right) _{ij}^{-1},\left( \left( I-\beta A\right) ^{-1}-I\right) _{ij}^{-1}D\right) \right) _{ij}^{-1}$	$\left( \max \left( D\left( A^2+\beta A^3\right) _{ij}^{-1},\left( A^2+\beta A^3\right) _{ij}^{-1}D\right) \right) _{ij}^{-1}$

Here *A* represents the adjacency matrix of the network, *I* is the identity matrix with size equal to the size of the matrix *A*, and *D* represents the diagonal degree matrix whose *i*th diagonal element is the degree of the *i*th node of the graph. Furthermore, $A^{-1}$ represents the inverse of the matrix *A*, while $A^{-1}_{ij}$ represents the element-wise inverse operation.

We commence by defining the global and quasi-local extensions of RA index. This index assigns more weight to the less connected neighbour. It can be shown that the RA index can be expressed in the form of matrix multiplication as $AD^{-1}A$, where *D* is the diagonal degree matrix whose *i*th diagonal element is the degree of the *i*th node. In order to define a global extension of the RA index, we not only consider the local paths between two nodes, but also consider the degrees of the nodes along the local paths. The global RA index is then defined as follows:

2 $$\begin{aligned} RA_G(u,v) = \beta A D^{-1} A +\beta ^2 A D^{-1} A D^{-1} A+\cdot \cdot \cdot = A\left( I-\beta D^{-1}A\right) ^{-1}-A. \end{aligned}$$

The global RA index, defined above, can be interpreted as follows: To predict the existence of a link between two nodes *u* and *v*, the global RA index considers all simple paths from *u* to *v*. Moreover, all the nodes on the paths contribute to the computation of the similarity index, where less connected nodes are assigned higher scores. As with Katz index, a damping parameter $\beta$ is used that assigns more weights to shorter paths. We also define a quasi-local extension of RA index that considers only the first two terms of the global RA index. Mathematically, this can be expressed as $RA_{QL}(u,v) = AD^{-1}A+\beta AD^{-1}AD^{-1}A.$

We define the global and quasi-local extensions of the AA in a similar way that we defined for the RA index. Note that, as mentioned earlier, the only difference between the AA and RA indices is that AA produces higher score values than RA for node pairs whose common neighbours have high node degree. This is achieved by taking the log of degrees of the common neighbours. The AA index can be expressed in the matrix form as $A(\log D)^{-1}A$, where $\log D$ is the diagonal degree matrix whose *i*th diagonal element is the log of the degree of the *i*th node.

Next, we define the global and quasi-local extensions of the remaining five similarity indices, i.e., SO, SA, LHN, HP, and HD. Note that all these five indices can be considered as modified versions of the CN index, that not only consider the the degrees of the common neighbours of the nodes *u* and *v*, but also take into account the degrees of the nodes *u* and *v* in one way or the other. Here we discuss the global and quasi-local extensions of SO index. The remaining indices can be extended in a similar way. By applications of simple matrix algebra, it can be shown that SO can be expressed in matrix form as follows:

3 $$\begin{aligned} SO(u,v) = \frac{2\left| \Gamma (u)\cap \Gamma (v)\right| }{|\Gamma (u)|+|\Gamma (v)|} = 2\left( D\left( A^2\right) _{ij}^{-1}+\left( A^2\right) _{ij}^{-1}D\right) _{ij}^{-1}, \end{aligned}$$

where $\left( A\right) _{i,j}^{-1}$ represents the element-wise inverse of the matrix A. Using matrix representation, we provide a straightforward global extension of SO by considering all the local paths between the nodes *u* and *v*, instead of only common neighbours. Therefore, we define the global extension of the SO as follows:

4 $$\begin{aligned} SO_G(u,v) = 2\left( D\left( \left( I-\beta A\right) ^{-1}-I\right) _{ij}^{-1}+\left( \left( I-\beta A\right) ^{-1}-I\right) _{ij}^{-1}D\right) _{ij}^{-1}. \end{aligned}$$

For the quasi-local extension of SO, we only consider paths up to length two. This index is defined as follows:

5 $$\begin{aligned} SO_{QL}(u,v) = 2\left( D\left( A^2+\beta A^3\right) _{ij}^{-1}+\left( A^2+\beta A^3\right) _{ij}^{-1}D\right) _{ij}^{-1} \end{aligned}$$.

The global and quasi-local indices of the remaining four local similarity indices (i.e., SA, LHN, HP and HD) can be defined in a similar way as we defined for the SO index. This is because the only difference between the SO index and each of these indices is the denominator has a different form. These extensions are reported in Table 1.

We conclude this section by discussing the time complexities of the global and quasi-local extensions of the the similarity indices discussed in this paper. We note that the key operations performed, when computing the global and the quasi-local extensions, are the two matrix operations, namely matrix multiplication and the matrix inversion. Both of these operations require cubic time in *N*, the number of nodes in the network. Therefore, the running time of both the quasi-local, as well as the global index, is bounded by $O(N^3)$. However, in practice, quasi-local index can be performed much faster as it takes into account only the information about the neighbours and the neighbours of the neighbours. Lü et al^33^ have demonstrated that the quasi-local extension provides a comparable performance to Katz index and it also requires less CPU time and memory space than Katz index. Furthermore, the computation of the global index requires a matrix inversion that is computationally very expensive (and can be unstable for large networks). In our experimental evaluation, we demonstrate that the quasi-local extensions of other local indices also result in a competitive performance, when compared to respective global extensions. Therefore, although the global extensions are effective for small and average-size networks, the quasi-local extensions are strong candidates for potential practical applications for large networks.

## Results and discussion

In this section we present the experimental evaluation results of the proposed methods on real-world datasets and compare the performance of local similarity indices with their global and quasi-local extensions.

### Datasets

To evaluate the performance of proposed and alternate methods, we have used various publicly available datasets from diverse domains, most of which are downloaded from KONECT^36^. A brief introduction of each of these datasets is given below. A summary of their topological properties is also presented in Table 2.

*Karate*^37^: A dataset (also known as the Zachary karate club) consisted of a karate club members, collected in 1977. The nodes of this network represent club members while the links represent ties between two members.
*US Roads*^6^: This network consists of 49 nodes and 107 links. The nodes represent the 48 contiguous states and the District of Columbia (Washington D.C.) of the USA and the links represent drivable roads between two nodes. This network includes all the states except the states of Alaska and Hawaii, which are not connected by land with the other states.
*Dolphins*^38^: A social network of bottlenose dolphins. The dataset consists of a set of links, each link representing frequent associations between dolphins.
*Train bombing*^39^: A dataset containing a list of 64 suspected terrorists who were believed to be involved in the Madrid train bombing on March 11, 2004. The nodes of the network represent the suspected terrorists, while the links between terrorists are established if the are friends or have co-participated in training camps.
*Caenorhabditis elegans (neurons)*^40^: This dataset consists of 279 neurons and 2990 links, including 1584 uni-directed and 1406 bidirected links. In our experiments, the direction was ignored resulting in a total of 2287 links.
*E. coli*^41^: A protein-protein interaction network of Escherichia coli that originally consisted of 424 nodes and 519 connections. We have considered the largest connected component (LCC) of the network having 329 nodes and 456 links.
*Network Science*^42^: A network of 1461 scientists working on network theory. In this network, the nodes represent scientists and a link is established between two scientists, if they are co-authors on the same paper. Similarly to the E.Coli dataset, we have only considered the LCC with 379 nodes and 914 links.
*Infectious*^43^: A network of 410 individuals who have attended exhibition, “infectious: stay away” in 2009 in Dublin. Here node represent individuals and a link represent face-to-face contact that was active for at least 20 seconds.
*Caenorhabditis elegans (metabolic)*^44^: This is the undirected metabolic network of the roundworm Caenorhabditis elegans, where nodes represent metabolites (e.g., proteins), and links represent the physical interactions between them.
*US Air*^45^: A network of direct flights among 500 US airports. The nodes represent airports and two nodes are connected if there is a direct flight between the corresponding airports.
*Email*^46^: An email communication network between individuals at the University Rovira i Virgili in Tarragona in the south of Catalonia in Spain. Here the nodes represent individuals and a link is established between two individuals, if one of the two users has sent at least one email to the other user. The direction and the frequency of the emails are ignored.
*Yeast*^47^: A yeast protein-protein interaction network, where each protein is a node and the interaction between them is represented by a link.

**Table 2:** Topological properties of the networks used in experiments.

Datasets	$\left| V\right|$	$\left| E\right|$	*C*	$\langle k\rangle$	$\langle d\rangle$	$\rho$	*H*
Karate^37^	34	78	0.588	4.588	1.204	0.139	7.769
US Roads^6^	49	107	0.507	4.367	2.082	0.091	4.935
Dolphin^38^	62	159	0.303	5.129	1.678	0.084	6.805
Train Bombing^39^	64	243	0.711	7.594	1.345	0.121	12.597
Neurons^40^	279	2287	0.337	16.394	1.218	0.059	25.916
*E. coli*^41^	329	456	0.222	2.772	2.421	0.008	12.314
Netscience^42^	379	914	0.798	4.823	3.021	0.013	8.021
Infectious^43^	410	17298	0.467	84.38	1.815	0.206	2.992
Metabolic^44^	453	4596	0.782	20.291	1.332	0.045	17.903
US Air^45^	500	2980	0.726	11.92	1.496	0.024	53.785
Email^46^	1133	5451	0.254	9.622	1.803	0.009	18.688
Yeast^47^	2375	11693	0.388	9.847	2.548	0.004	34.223

|*V*| and |*E*| are the number of nodes and links respectively. *C* is the clustering coefficient. $$\langle k \rangle $$ and $$\langle d \rangle $$ are average degree and average path length. Finally $$\rho $$ denotes the density of the network while *H* is the heterogeneity defined as $$H = \frac{\langle k^2 \rangle }{\langle k \rangle ^2}.$$

### Evaluation metric

In order to assess and compare the performances of the local similarity indices and their corresponding global and quasi local extensions, we computed their accuracies using the area under the receiver operating characteristic metric *AUC*^48^. Consider a simple network $G = (V,E)$. Her we refer to the set *E* as the set of observed links. Let $E'$ represents the set of nonexistent links in the network. In other words, $E' = \{(u,v): u,v \in V, (u,v) \notin E\}$. We note that, if *U* represents the set of all possible $\frac{|V|\left( |V|-1\right) }{2}$ edges that *G* can have, then $E' = U \setminus E$. In order to evaluate the prediction algorithm’s performance, the set of observed links, *E*, is randomly divided into two disjoint sets, namely, a training set $E^T$ and a probe set $E^P$. Since $E^T$ and $E^P$ are disjoint, the two sets form a partition of the set *E*, i.e., $E = E^T \cup E^P$, and $E^T \cap E^P = \phi$. The information in $E^T$ is used to predict missing links while the information in $E^P$ is used to evaluate the performance of the prediction algorithm. To estimate the accuracy of the prediction algorithm, we compute their AUC values. In our case the metric AUC can be interpreted as the probability that a randomly chosen link in $E^P$ gets higher score than a randomly chosen link in $E'$. If among *n* independent comparisons, $n'$ is the number of times a missing link has higher score than a non-existent link, and $n''$ is the number of times a missing link and a nonexistent link having the same score, then the *AUC* is defined as

$$\begin{aligned} AUC = \frac{n'+0.5n''}{n}. \end{aligned}$$

We note that the value of AUC should be about 0.5, if all the link scores are randomly generated according to an independent identical distribution. Therefore, a value greater than 0.5 indicates how well the prediction algorithm performs when compared to pure chance.

### Experimental results

**Figure 1:**
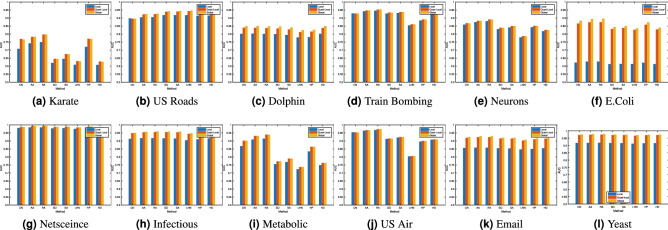
Bar chart comparison of the accuracies, measured by AUC values, resulting from the application of different methods. The difference between the AUC values of the local indices and their respective extensions is significant in most cases.

In order to assess the performance of the global and quasi-local similarity indices and compare it with the local similarity indices’ performance, we have randomly divided the set of observed links of the network, *E*, into two sets, namely, a training set $E^T$ and a probe set $E^P$. In our first experiment, 90% of the observed links were contained in the training set while the remaining 10% were used for the probe set. The performance of all the similarity indices were evaluated using the same training and probe sets. For the quasi-local and the global indices, the value of parameter $\beta$ was set to 0.001. The experiment was repeated 100 times, and in each run an independent random sampling of the observed links was performed. The average accuracies (along with standard deviations) of all the 100 runs are reported in Table 3. Figure 1 presents a visual representation of these results.

**Table 3:**
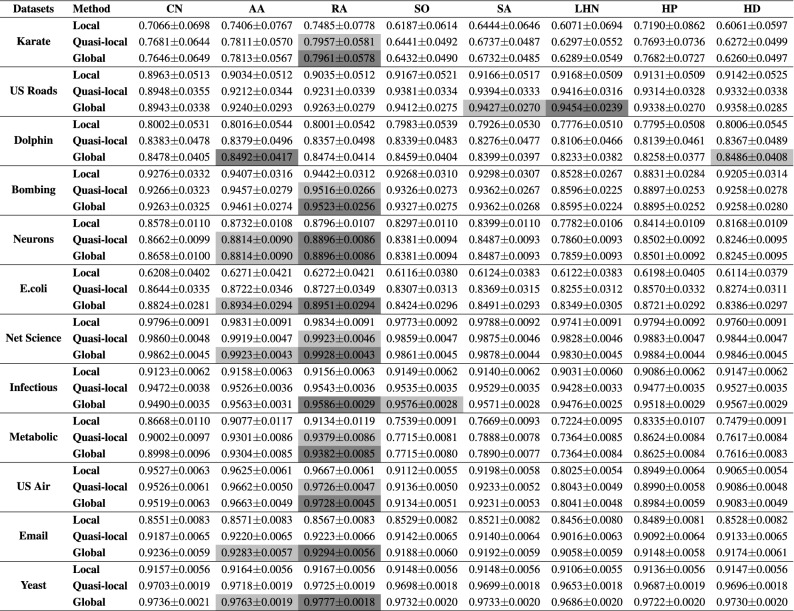
The prediction accuracy of the local, quasi-local, and the global indices for each method, measured by AUC.

Each experiment was executed 100 times with independent random network division of the training set and the probe set and the average value along with standard deviations of all the 100 runs are reported. The cells highlighted in gray colour present the best performance obtained while the cells highlighted in light-grey colour present the second best performance.

It is evident from the results that both the local and the global extensions can increase the prediction accuracy of the corresponding local indices. These global and quasi-local extensions not only result in high accuracies, but the accuracies’ variations are also low when compared to the variations observed in local indices. We note that for some of the networks presented in Table 2, such as E.Coli network, the performance has been significantly increased, when longer paths are considered, whereas for other networks, the difference is not very significant. This improvement in performance may be attributed to the topological properties of the network, in particular, the average clustering coefficient and the density of the network. For a sparse network with a low clustering coefficient, it is unlikely that a similarity index, computed purely based on the degree statistics of immediate neighbour, will predict links with higher accuracy. From the statistics of networks presented in Table 2, one can see that the E.Coli dataset is very sparse and has the lowest clustering coefficient. Train bombing, on the other hand, is denser with a high clustering coefficient. Consequently, the increase in performance for the E.Coli dataset is around 25%, whereas for the Train bombing dataset, the performance has increased by less than 1%. Further information about the difference between the AUC values of global and quasi-local extension from their respective local index is presented in supplementary material (Table S1). In terms of comparison among the global and the quasi-local extensions of different indices, we observed that the path-based extensions of the RA index outperform all the alternative methods (including Katz index) on most of the datasets. Furthermore, the difference between the performances of the global and the quasi-local extensions of the RA index is also not very significant in most cases. Finally, it is also worth noting that the quasi-local extension of both the RA index and AA index always give superior performance when compared to local path index (a quasi-local extension of CN index). These results suggest that by incorporating the degree information of nodes on local paths, the prediction accuracies of local indices can be significantly improved.

**Figure 2:**
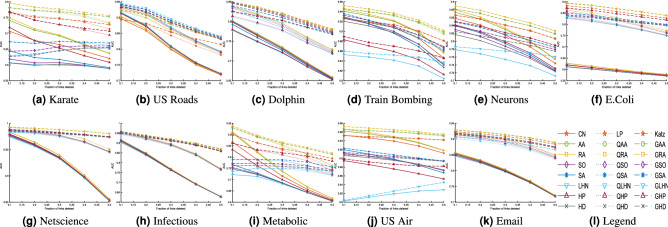
The prediction accuracy of the proposed and the alternative methods, measured by AUC, with different split of training and probe set. As with the previous experiments, each value is obtained by averaging over 100 executions of experiments with independently random divisions of training set and probe set.

To further investigate the performances of the global and quasi-local extensions and compare it to local indices, we evaluate the classification accuracy with different partitioning sizes of training and probe sets. For this purpose, we choose different sizes of the probe sets as 20%, 30%, 40%, 50% respectively. We have chosen eleven datasets in this experiment. For each split, we have computed the accuracies of the local indices, and both their global and quasi-local extensions. To visualise and compare those results, we have plotted the average accuracies of 100 independent runs of each experiment (with independent random splitting of *E* into $E^P$ and $E^T$) in Figure 2. For comparison purpose, we have also included the results of the previous experiment, where we have chosen the size of the probe set as 10%, in the plot of Fig. 2. Note that, for large networks, the time required to compute AUC significantly increase with increase in probe size. Therefore, we have excluded the yeast dataset in this experiment.

There are a number of important observations that can be made from the results plotted in Fig. 2. Firstly, the performance of all the methods generally decreases with the increase in training size. This is obvious, as with the decrease in training size, we have less information available to predict links. This reduces the performance of the prediction algorithm. Secondly, in most case, when the structural error is very high, the local similarity indices suffer from low performance, while the global extensions can still give reasonably better performance. This is because of the fact that when we delete more links from the network, the local topology of network is considerably changed. In such cases, the global similarity indices, that take into account the overall topology of the network, can outperform the local/quasi-local indices. As expected, when the structural error is high, the performance of a quasi-local index is always higher than the corresponding local index but less than the corresponding global index. Furthermore, the global extension of RA index usually outperforms all the other methods including Katz index for different partition sizes. Finally, for two datasets, namely Karate and US Air, we note that the prediction accuracy of some indices increases with increase in the size of probe test. This may be due to the fact that some link prediction algorithms, such as LHN, SA and SO, depend upon the degrees of query nodes. With more edges deleted from the network, such indices may predict links with higher accuracy for some specific datasets.

In order to assess the performance of the proposed link prediction indices, we compare their accuracies with some state-of-the-art link prediction algorithms. For this purpose, we have applied five alternative methods, namely, the MFI (Matrix-Forest Index)^27^, the LO (Linear Optimisation)^28^, the CND (Common Neighbour and Distance information)^24^, the CAR-based indices proposed by Cannistraci et al.^20^, and the PA (Preferential Attachment)^16^ across all the twelve datasets we have used for performance assessment. The AUC values, obtained from the application of all these methods, are presented in Table 4. For the CAR-based indices, we have only reported the accuracies of the CAR-based extension of the RA index (CRA), as we observed that it outperforms all the other car-based indices. As discussed earlier, since the quasi-local extension of the RA index can be efficiently computed and gives comparable performance, for comparison purposes, we have also included its accuracies in the table. The results show that the $\hbox {RA}_Q$ gives best or close to best performance when compared to alternate methods for all the datasets that were used for performance assessment. Additionally, it can also be verified from the results that the $\hbox {RA}_G$ outperforms all the alternate methods. To investigate further, we have also computed the precision of prediction accuracies for all the methods. The results are presented in the supplementary material (Fig. S1).

**Table 4:**
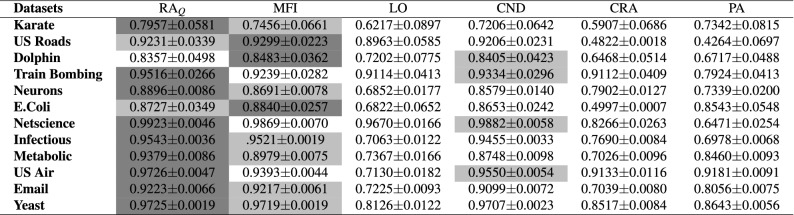
The prediction accuracies of our proposed method as well as of the state-of-the-art methods we bench-marked it against, measured by AUC.

Each experiment was executed 100 times with independent random network division of the training set and the probe set and the average value of all the 100 runs are reported.

**Figure 3:**
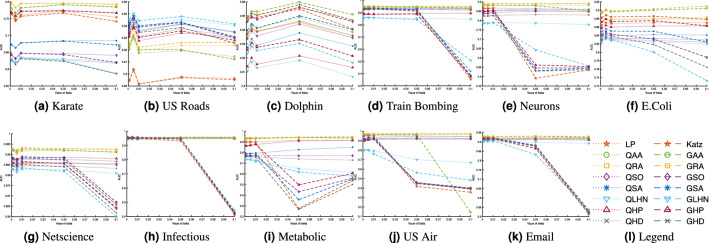
The prediction accuracy of the proposed and the alternative methods, measured by AUC, with different values of beta. As with the previous experiments, each value is obtained by averaging over 100 executions of experiments with independently random divisions of training set and probe set.

In our final experiment, we investigate the performances of the global and quasi-local indices by varying the values of the parameter $\beta$. We have selected five different values of the parameter $\beta$, i.e., 0.001, 0.005, 0.01, 0.05, and 0.1. The resulting accuracies for different datasets are plotted in Fig. 3. These results suggest that both the global and quasi-local indices perform well for small values of the parameter $\beta$. The prediction accuracy generally decreases when the value of the parameter $\beta$ increases. This is due to the fact that for higher values of $\beta$, the longer paths are assigned more weights. This difference is significant for the global indices as it also considers paths with lengths greater than two. Note that a sudden drop in the performances of the global indices is due to the convergence problem of the global indices. In such cases, the performance can be approximated by expanding the series and considering the first few terms.

## Conclusion

In this paper, we have proposed quasi-local and global extensions of local similarity indices that are used to predict the likelihood of existence of a link between two nodes in a network. This was achieved by considering local paths of different lengths and the information of the nodes on those local paths. We have also provided vectorised implementation of all the local methods and their proposed extensions. Experimental results on publicly available datasets demonstrate that both the global and the quasi-local extensions can increase the prediction accuracies of local methods. The performance of the proposed similarity indices was also reviewed with respect to different sizes of the probe sets and varying values of the parameter $\beta$. In both these cases, our proposed similarity indices achieved higher performance. The proposed method was applied to various domains including chemical networks, biological networks and social networks. In terms of future work, we plan to extend the work presented here to bipartite networks such as drug-target interaction networks. Note that, the experiments performed in this paper were limited to only simple networks, whose edges are unweighted and undirected. However, the proposed similarity indices can be easily extended to more complicated cases such as directed networks or weighted networks.

## Supplementary information

Supplementary Information.
