# Supplementary material for: Path-based extensions of local link prediction methods for complex networks
Source: Sci Rep. 2020 Nov 16;10:19848. doi: 10.1038/s41598-020-76860-2 (PMC7670409; doi:10.1038/s41598-020-76860-2)
Supplement: Supplementary file 1 — Supplementary Information. [file 41598_2020_76860_MOESM1_ESM.pdf]

Supplementary Material: Path-based Extensions of Local Link Prediction Methods for Complex Networks

Furqan Aziz[1,2,3,4*], **Haji Gul**[5], **Irfan Uddin**[6], **and Georgios V. Gkoutos**[1,2,3,4,7,8]

[1]Centre for Computational Biology, University of Birmingham, B15 2TT, Birmingham, United Kingdom
[2]College of Medical and Dental Sciences, Institute of Cancer and Genomic Sciences, University of Birmingham, B15 2TT, Birmingham, United Kingdom
[3]Institute of Translational Medicine, University Hospitals Birmingham NHS Foundation Trust, B15 2TT, Birmingham, United Kingdom
[4]MRC Health Data Research UK (HDR), United Kingdom
[5]City University of Science and Technology, Peshawar, Pakistan
[6]Kohat University of Science and Technology, Kohat, Pakistan
[7]NIHR Experimental Cancer Medicine Centre, B15 2TT, Birmingham, United Kingdom
[8]NIHR Surgical Reconstruction and Microbiology Research Centre, B15 2TT, Birmingham, United Kingdom
[*]f.aziz@bham.ac.uk

Datasets	Method	CN	AA	RA	SO	SA	LHN	HP	HD
**Karate**	**Qausi-local**	6.15	4.05	4.72	2.54	2.93	2.26	5.03	2.11
	**Global**	5.8	4.07	4.76	2.45	2.88	2.18	4.92	1.99
**US Roads**	**Qausi-local**	-0.15	1.78	1.96	2.14	2.28	2.48	1.83	1.9
	**Global**	-0.2	2.06	2.28	2.45	2.61	2.86	2.07	2.16
**Dolphin**	**Qausi-local**	3.81	3.63	3.56	3.56	3.5	3.3	3.44	3.61
	**Global**	4.76	4.76	4.73	4.76	4.73	4.57	4.63	4.8
**Bombing**	**Qausi-local**	-0.1	0.5	0.74	0.58	0.64	0.68	0.66	0.53
	**Global**	-0.13	0.54	0.81	0.59	0.64	0.67	0.64	0.53
**Neurons**	**Qausi-local**	0.84	0.82	1.00	0.84	0.88	0.78	0.88	0.78
	**Global**	0.8	0.82	1.00	0.84	0.88	0.77	0.87	0.77
**E.coli**	**Qausi-local**	24.36	24.51	24.55	21.91	22.45	21.33	23.72	21.6
	**Global**	26.16	26.63	26.79	23.08	23.67	22.27	25.23	22.72
**Net Science**	**Qausi-local**	0.64	0.88	0.89	0.86	0.87	0.87	0.89	0.84
	**Global**	0.66	0.92	0.94	0.88	0.9	0.89	0.9	0.86
**Infectious**	**Qausi-local**	3.49	3.68	3.87	3.86	3.89	3.97	3.91	3.8
	**Global**	3.67	4.05	4.3	4.27	4.31	4.45	4.32	4.2
**Metabolic**	**Qausi-local**	3.34	2.24	2.45	1.76	2.19	1.4	2.89	1.38
	**Global**	3.3	2.27	2.48	1.76	2.21	1.4	2.9	1.37
**US Air**	**Qausi-local**	-0.01	0.37	0.59	0.24	0.35	0.18	0.41	0.21
	**Global**	-0.08	0.38	0.61	0.22	0.33	0.16	0.35	0.18
**Email**	**Qausi-local**	6.36	6.49	6.56	6.13	6.19	5.6	6.03	6.05
	**Global**	6.85	7.12	7.27	6.59	6.71	6.02	6.59	6.46
**Yeast**	**Qausi-local**	5.46	5.54	5.58	5.5	5.51	5.47	5.51	5.49
	**Global**	5.79	5.99	6.1	5.84	5.85	5.8	5.86	5.83

Table S1. The increase/decrease in performances of the quasi-local and global indices when compared to their respective local indices. We note that the difference is significant in some cases. For examples, for the E.coli dataset, the increase in accuracies is more than 20%. As discussed in the paper, this improvement in performance may be attributed to the topological properties of the network, in particular, the average clustering coefficient and the density of the network.

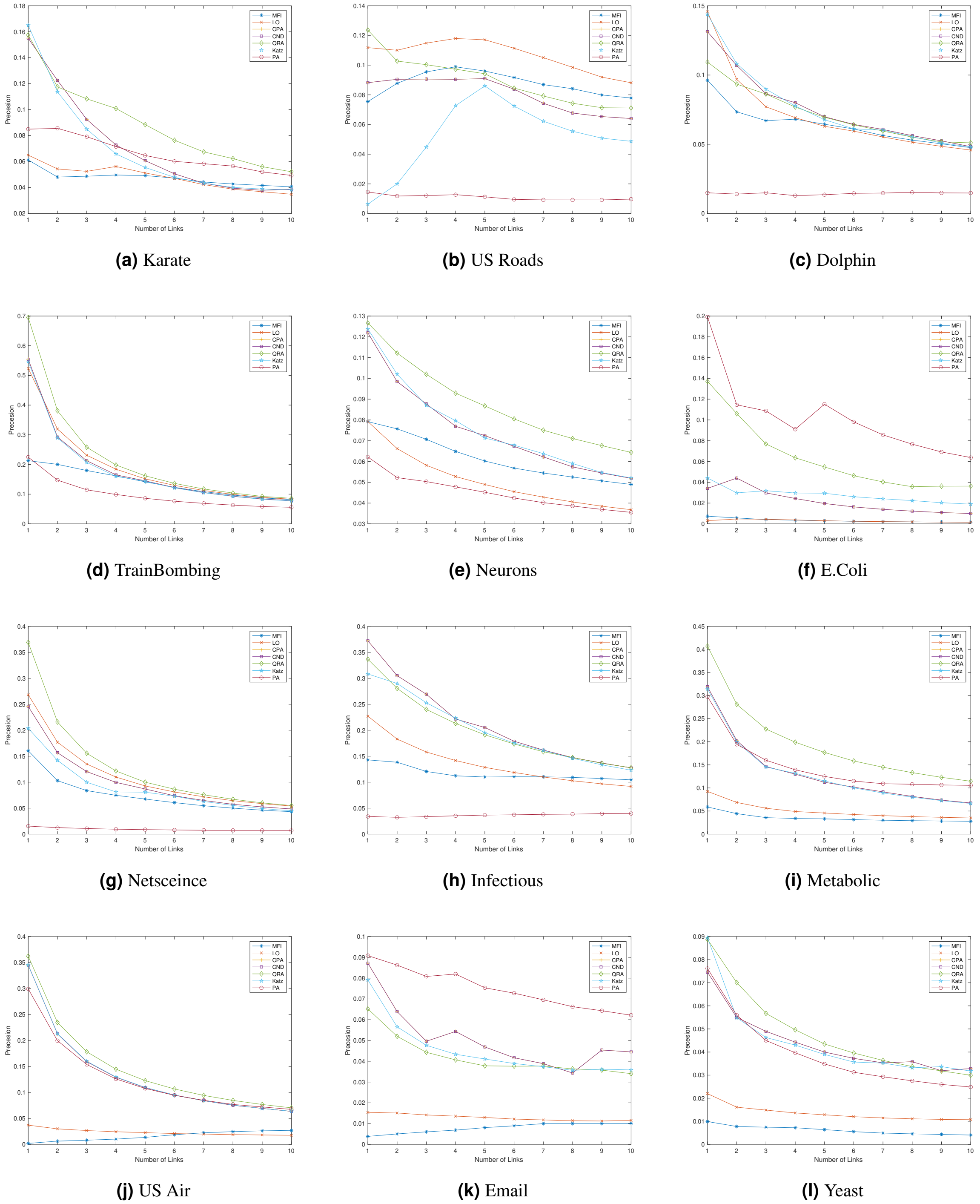

(a) Karate **(b)** US Roads **(c)** Dolphin

(d) TrainBombing **(e)** Neurons **(f)** E.Coli

(g) Netsceince **(h)** Infectious **(i)** Metabolic

(j) US Air **(k)** Email **(l)** Yeast

Figure S1. Accuracies of different methods as measured by Precision. To compute precession, the non-observed links are sorted in decreasing order. If L_r links are correctly predicted links among top L links, then the precision is defined as $Precision = \frac{L_r}{L}$. We note that the quasi-local extension of the resource allocation index give higher precision values on most dataset (and comparable performance on the other dataset).
